# The three-domain impact framework for characterizing impact of patient involvement in health technology assessment

**DOI:** 10.1017/S0266462324000400

**Published:** 2024-11-11

**Authors:** Veronica Lopez Gousset, Aline Silveira Silva, Anke-Peggy Holtorf, Ana Toledo-Chávarri, Ann Single

**Affiliations:** 1 VLG Consulting, Paris, France; 2 Research Group on Access to Medicines and Responsible Use (AMUR–UnB), University of Brasilia, Brasilia, Brazil; 3 Health Outcomes Strategies, Basel, Switzerland; 4 University of Utah, College of Pharmacy; 5 Canary Islands Health Research Institute Foundation (FCIISC), El Rosario, Spain; 6 Patient Voice Initiative, Sydney, Australia; 7 Health Technology Assessment International Patient and Citizen Involvement Group (HTAi-PCIG), Edmonton, Canada

**Keywords:** patient involvement, health technology assessment, impact, qualitative evaluation

## Abstract

**Objectives:**

Evaluating the impact of patient involvement in health technology assessments (HTA) may help improve practices and avoid ineffective activities. Evaluation, however, continues to be infrequent, inconsistent, and often only relates to process quantity or quality. The Patient and Citizen Involvement in HTA Interest Group (PCIG) within Health Technology Assessment International set out to contextualize this impact to support evaluation.

**Methods:**

Given the lack of established methodology to measure impact, the team performed a qualitative analysis of first-hand accounts about perceived changes in HTA due to involvement of patient stakeholders. A questionnaire was developed, piloted, and rolled out to collect personal perspectives from stakeholders with relevant experience. The stories were analyzed in the aggregate to identify themes in the data.

**Results:**

From January 2019 to September 2021, twenty-four responses were collected through PCIG’s network. Responses (including one joint industry-HTA body submission) came from patient representatives (12), HTA bodies (11), and industry representatives (2) from North America (5), South America (3), Europe (13), and Asia Pacific (3). Based on themes commonly reported, a three-domain framework for evaluating impact is proposed: impact on basis of HTA result or recommendation, impact on HTA body, and impact on patient participants. The framework includes components under each domain to support reporting.

**Conclusions:**

Using the Three-Domain Impact Framework may be useful in identifying, evaluating, and communicating the value of patient involvement in HTA. Enhancing and increasing reporting practices may improve transparency and facilitate process improvements for meaningful integration of patient stakeholders into HTA appraisals across jurisdictions.

## Introduction

Health technology assessment (HTA) bodies often have processes to consider patients’ needs, preferences, and experiences in their assessments by using participation or patient-based evidence (1). However, the impact of involving patients can be unclear and may be described differently across stakeholder groups (2,3). Evaluating the impact of patient involvement in HTA is essential to demonstrate its effectiveness for both patient groups and HTA bodies, improve efficiency, and develop good practices (4). Yet, the impact of patient involvement is difficult to measure because HTA includes deliberation on multiple sources of evidence and insight (5) and being transparent about these deliberations can be challenging (6,7). There is still ambiguity around how to best characterize and report on this impact across HTA bodies and processes.

Members of the Patient and Citizen Involvement in HTA Interest Group (PCIG) within Health Technology Assessment International (HTAi) set out to define a simple approach to categorize the impact of patient involvement in HTA which might support increased transparency and improve the practice of consistent identification, reporting, and evaluation. This work builds on two previous projects conducted by PCIG, including an environmental scan of how HTA bodies evaluate their patient involvement initiatives (3) and case reports by HTA bodies on the impact of patient involvement in improving processes and recommendations (8).

This study analyzes how the impact of patient involvement across HTA processes is perceived by the different stakeholders involved and identifies elements that add value and those that can be improved. Based on the findings, a three-domain framework is proposed to enhance the identification, evaluation, and reporting of patient involvement in HTA, support cross-country exchange and advancement across jurisdictions, and manage expectations for the impact of patient involvement in HTA.

## Methods

Because impact means different things to different people, the team performed a qualitative study of first-hand accounts (stories) collected through an online questionnaire about perceived changes in HTA due to the involvement of patient stakeholders. For the purposes of this analysis, we defined patient involvement as any form of participation of patients during the evidence collection, review, and deliberation process. We defined impact as a change perceived to be the result of patient involvement in HTA.

To collect this information, a series of open-ended questions guided stakeholders to share their perspectives and experiences through storytelling. The questionnaire included an informed consent section, as well as background and categorization questions. A first draft of the questionnaire template was piloted in early 2019. Two stories were collected, and a second draft was created to improve the clarity of the questions based on user feedback. The second draft was piloted in March 2019, and three additional stories were gathered. This second collection of stories confirmed their informative value and led to the third and final version of the questionnaire launched in December 2020 (Supplementary File 1).

This questionnaire was disseminated using a convenience sample of HTA practitioners, patient representatives, and industry stakeholders with experience in patient involvement in HTA. Recruitment occurred through promotion across PCIG (distribution list, e-bulletin), HTAi events (annual meetings, workshops, webinars) and personal invitations. Nineteen additional stories were collected through September 2021. All pilot responses were included in the final data set as changes to the template only improved readability and did not affect content.

An adapted thematic analysis on the qualitative content of the twenty-four stories was used to contextualize the impact of patient involvement in HTA processes (9). First, the authors read nine stories to identify initial themes in the data. These submissions were split across the five authors (APH, AS, ASS, ATC, VLG) so that each story was reviewed by two authors to ensure consistency of interpretation. The team then developed a code book from the initial themes identified. Six additional stories were then randomly selected for coding by two additional authors (APH, ATC) to test the reliability of the code book. One of the authors (VLG) applied the codifiers to all twenty-four stories collected from January 2019 to September 2021 and developed additional codifiers as needed. The group reviewed and agreed upon any differences in the coding across authors. Lastly, the domains of impact were generated manually, using Microsoft Excel to organize the data, by clustering the codifiers based on common patterns and connections.

## Results

From January 2019 to September 2021, twenty-four impact stories (including one joint industry-HTA body submission) were collected from patients or health consumers and their representatives who have provided input into assessments (twelve), people working in HTA bodies or researchers commissioned to do assessments (eleven), and industry employees who prepared submissions (two). These stories were related to the assessment of medicines (fifteen), medical devices (three), procedures (one), and development of HTA guidelines (three). Three entries reported on a group of evaluations or the general work of an HTA body. The most common therapeutic areas across the stories submitted were oncology and neurology (five stories each), followed by respiratory and metabolic conditions (three each). Lastly, these stories were collected across Europe (thirteen), North America (five), South America (three), and Asia Pacific (three), with the most represented countries across all geographies being England (seven), Canada (four), Australia (three), and Brazil (three). See Table 1 for an overview of all entries.

**Table 1.** Overview of twenty-four stories collected from January 2019 through September 2021. Stories are organized by stakeholder type in the order in which they were received

Response number	Type of respondent *self-reported category	Country	Type of technology	Therapeutic area	Date of HTA review
P1	Patient organization representative	Australia	Medicine	Respiratory rare	2016
P2	Patient organization representative, researcher; Patient expert*	England, United Kingdom	Medicine	Metabolic rare	2016
P3	Patient organization representative, researcher; Patient expert*	Germany	Medicine	Metabolic rare	2016–17
P4	Patient organization representative	England, United Kingdom	Medicine	Oncology	2006
P5	Patient organization representative; patient/health consumer	Canada	Medicine	Oncology	Multiple
P6	Patient/health consumer	Australia	Medicine	Oncology	2017–19
P7	Patient organization representative	England, United Kingdom	Medicine	Neurology rare	2019
P8	Patient organization representative	England, United Kingdom	Medicine	Oncology	2020
P9	Patient organization representative	Scotland, United Kingdom	Procedure	Neurology	2019
P10	Patient organization representative	Brazil	Medicine	Neurology	2015–21
P11	Patient/health consumer	Canada	Multiple	Multiple	2019 – present
P12	Patient organization representative, researcher; Patient/health consumer	Brazil	Medicine	Respiratory rare	2020
H1	HTA staff member	Scotland, United Kingdom	Medical device	Metabolic	Not provided
H2	HTA staff member	England, United Kingdom	Medicine	Immunology	2017
H3	Researcher (Commissioned to do HTAs for Provincial Ministry of Health)	Canada	Multiple	Diverse	Ongoing
H4	HTA staff member	Brazil	Multiple	Multiple	Ongoing
H5	HTA staff member	England, United Kingdom	Medicine	Neurology rare	2018
H6	HTA staff member	Unites States of America	Medicine	Immunology and hematology	2020–21
H7	HTA staff member	Canada	Medical device; guideline	Cardiovascular and infection	2017, 2019
H8	HTA staff member	Spain	Medical device	Congenital defect	Not provided
H9	HTA staff member	Wales, United Kingdom	Guideline	Neurology	2020
H10	HTA staff member	Wales, United Kingdom	Guideline	Optometry	2021
H11/I1	HTA staff member and Industry staff (joint submission)	England, United Kingdom	Medicine	Respiratory rare	2018
I2	Industry staff	Australia	Medicine	Oncology	2017–20

The first and most important finding from the analysis of responses is that impact is reported across at least three domains:

- Impact on basis of HTA result or recommendation.
- Impact on HTA body.
- Impact on patient participants.

Each domain comprises multiple ways in which patient involvement is perceived to impact the various HTA applications, including scientific advice, assessment, appraisal, and post-HTA data collection. See Table 2 for an overview of the codifiers under each domain and the corresponding stories that reported each type of impact.

**Table 2.** Codifiers under each domain and corresponding stories that reported each type of impact

Domain of impact	Codifiers per domain	Total number of stories that reported this type of impact out of 24 stories (corresponding responses)
Impact on basis of HTA result or recommendation	*Data interpretation* – Improves data interpretation by contextualizing/reframing/providing (alternate) interpretation of evidence presented/data based on realities of living with condition; supports/counters claims made by other stakeholders	19 (P1, P2, P3, P4, P5, P7, P8, P9, P12, H1, H2, H3, H4, H5, H7, H8, H9, H10, H11/I1)
	*Patient and caregiver lived experience* – Improves understanding of what it is like to live with or care for someone with the condition; unfiltered, nonmediated experience	17 (P1, P2, P3, P5, P7, P8, P9, H1, H2, H4, H5, H6, H7, H8, H9, H10, H11/I1)
	*Patient needs* – Increases awareness of patient community unmet needs, including treatment needs	12 (P2, P5, P6, P7, P9, H1, H2, H6, H7, H9, H10, I2)
	*New data consideration* – Acknowledges additional information/outcome measures not captured in literature or clinical trials, for example, contribution of patient-led research (patient preference studies, real-world evidence studies)	11 (P2, P5, P9, H2, H4, H5, H6, H7, H8, H9, H11/I1)
	*Recommendation* – Change of HTA recommendation or appeal decision direction due to patient input	10 (P2, P3, P4, P6, P10, P12, H4, H7, H9, H10)
	*Subpopulations* – Increases awareness of inequalities, diversity of patient populations, and special needs of subpopulations	6 (P2, P9, P11, H4, H6, H10)
	*Cost data* – Acknowledges cost data from patient community, for example, financial implications for patients and health economic considerations	5 (P5, P9, H2, H7, H8)
	*Data limitations* – Recognizes limitations of existing data and implications of missing data	4 (P3, H5, H6, H9)
	*Patient acceptability of technology* – Recognizes practical implications of treatment (uptake, adherence), acceptability by patient population (benefit/risks, tolerability), and consequences for implementation	3 (P5, H4, H7)
Impact on HTA body	*HTA staff awareness of patient involvement importance* – Increases understanding of value of involving patient perspective in HTA process	7 (P1, P11, H1, H4, H6, H10, H11/I1)
	*Purpose-driven HTA* – Reminds HTA personnel of the final consumer and the reason why they do the work they do	5 (P8, P11, H7, H9, H10)
	*HTA engagement culture* – Increases culture of patient involvement at the organizational level	4 (P11, H1, H7, H9)
	*Perceptions of patients as equal partners* – Supports reframing of patient involvement in HTA from patients as a source of experiences/perspectives to valuable, trusted partners able to meaningfully contribute to discussion	4 (P4, P11, H9, H10)
	*Direct contact/first-hand validation* – Provides HTA committee members access to first-hand, uninterpreted patient experience and ability to ask questions live	3 (P1, H2, H9)
	*HTA process improvement* – Acknowledges suggestions on how HTA processes for patient involvement (and generally) can be improved from patient perspective	2 (H1, P11)
Impact on patient participants	*Acknowledgement* – Engenders feeling of being heard, valued, and other positive emotional impact	3 (P1, P3, P9)
	*Co-construction* – Provides ability for patient stakeholders to contribute to access of new treatments and make a difference in their treatment course and that of future patients	3 (P4, P6, P10)
	*Patient participation in HTA* – Increases culture of participation in future HTA processes at the organizational and individual level; sets precedent for other patient communities to participate in HTA processes	2 (P1, P10)
	*Patient awareness of HTA* – Increases patients’ knowledge and understanding of HTA and its process	1 (P1)
	*Patient recommendation acceptance* – Increases acceptance of the HTA recommendation as a result of better understanding the purpose of HTA and its process	1 (P1)

In addition, the responses revealed three primary areas for improvement:

- Transparency and expectation-setting.
- Training and data readiness.
- Systems and processes.

See Table 3 for an overview of the improvement codifiers and the corresponding source stories.

**Table 3.** Codifiers under each area for improvement and corresponding stories that reported these opportunities to improve

Area for improvement	Codifiers per area	Total number of stories that report this type of improvement out of 24 stories (corresponding responses)
Transparency and expectation-setting	Increase transparency of HTA patient involvement processes (who is invited, how will testimonies be considered, opportunities for participation, etc.)	9(P1, P2, P6, P7, P8, P10, P12, H6, I2)
	Improve feedback as part of submission process (expected follow up posthearing, feedback on testimonials/participation, feedback process)	5(P1, P2, P9, P12, H10)
	Provide guidance to support effective patient participation in the HTA process (instructions for participating, setting, duration, expectations, audience, etc.)	2(P1, H6)
Systems and processes	Encourage involvement and/or more systematic involvement across different stages of HTA process, including direct interactions	7(P5, P6, H1, H4, H7, H10, I2)
	Improve the systematic review of patient involvement and feedback processes	4(P7, P10, P12, H4)
	Publish and promote opportunities to participate in HTA process widely	3(P1, P12, H4)
	Increase representativeness of patient participants (e.g., through patient-based evidence)	3(P3, P5, H11/I1)
	Improve submission templates for patient community providing input	1(P5)
Training and data readiness	Increase communication of the value of patient participation to HTA leadership (internal alignment on purpose/objectives for involving patients) and train HTA personnel on what is appropriate and reasonable to ask of patients (sensitivity training)	7(P1, P2, P5, P8, P10, H7, H9)
	Increase guidance and training of patients on how to participate effectively in HTA processes (what it is, purpose, evaluation criteria)	4(P1, P11, H3, I2)
	Provide indications to improve capabilities and capacity of stakeholder community for generating robust data/evidence	4(P4, H2, H5, H9)
	Increase awareness among other stakeholders of importance of patient involvement, for example, medical community participating in HTA processes	2(H7, H10)
	Develop buddy system where patients previously participating in HTA processes can partner with patients new to the process to manage expectations	1(P1)

### Domains of impact

#### Impact on basis of HTA result or recommendation

This domain covers the impact on the evidence base and deliberation process that forms the basis of the HTA result or recommendation; it highlights ways in which patient involvement can contribute to broadening the evidence base, support contextualization, and strengthen the quality of deliberations and the appraisal process alongside other stakeholders.

The most commonly reported example of impact identified under this domain was improved *data interpretation.* This type of impact, which featured across nineteen of the twenty-four stories, focuses on instances where the patient perspective helped to contextualize, reframe, or otherwise provide an interpretation of evidence reviewed as part of the assessment. For example, understanding the experience of living with the condition may help data interpretation, support or counter claims made by others, or identify outcomes of importance to patients. In the joint HTA body-industry submission respondents cited, “In the absence of this patient input, the clinical expert view would likely have prevailed during the discussion… the patient input supported the alternative view, consistent with findings from the qualitative research” (response H11/I1). Another HTA respondent shared that “Information provided by the patient submission supported the company’s statements regarding the value placed on the therapy by patients and the degree of improvement in quality of life after successful treatment. It would have been near enough impossible for the Committee to get a feel for this without such direct experience” (response H2). Many HTA respondents emphasized that the meaningfulness of small clinical differences can be accentuated through patient input, “this helped to put the clinical and economic evidence into context” (response H9); “[patient input] provided a reminder of how small benefits may be transformational” (response H5); “this contributed to alleviating some of the doubts members had surrounding gaps in the clinical evidence” (response H10).

Relatedly, other commonly reported examples of impact under this domain were contributions from patient representatives that increased the understanding of the *patient experience* (mentioned in seventeen out of twenty-four stories) and highlighted *patient needs* (twelve mentions). Specifically, patient participants provided an unfiltered perspective of living with the condition, how it was affecting the (quality of) life for patients and caregivers, and insights into how the current treatments were not meeting their needs. For example, a patient organization contributed “personal perspectives, the reality of living with [condition], the symptoms and challenges it poses on a day-to-day basis and the reasons patients want to proceed with [procedure] rather than medication” (response P9); “the patient group submission brought a new dynamic to the meeting and showed the reality of what happens for someone who has [condition] and how [device] can help” (response H1). Without patient involvement, “the committee would have been less aware of the merits of the drug and missed some of the success stories amongst patients” (response P6).

The presentation of *new data* for the committee’s consideration (eleven mentions) and the demonstration of *data limitations* (four mentions) were two additional ways patient involvement made a difference during deliberation. For example, patient organizations doing research to present data to HTA bodies, “the survey the patient organization conducted… was particularly helpful… [in] underpin[ning] the willingness to travel and the likely uptake of the treatment” (response H2), or highlighting gaps in existing data, “Patient testimony at the meeting indicated that there were other aspects of the disease which were not captured in the model; indicating that QALY measurement may not be capturing full benefit [and that] impact on carers wasn’t properly captured” (response H5).

Other types of contributions under this domain included presentation of *cost data* (five mentions), “relevant aspects related to the uptake of the technology would have been disregarded, such as the costs that are [borne] by the patients and their families” (response H3), stressing *patient acceptability* (three mentions), including patient perspectives on the risk/benefits tradeoffs of new therapies; and shedding light on inequalities or special needs of *subpopulations* (six mentions), “[patient involvement] allowed to include information about how African Americans are more impacted by the disease” (response H6).

Lastly, ten out of twenty-four stories mention some direct impact of patient involvement on the *recommendation* itself, including changes in the HTA recommendation or support for an appeal process. “The Appeal Panel… was convinced by the argument and the patient input confirming that under treatment they have an almost normal life and upheld the Appeal point that the benefit of the treatment is not ‘small’” (response P2); “Patient participation is directly related to the recommendation that changed from initial recommendation to disinvest… to not to disinvest” (response P10). In contrast, four stories (P7, P8, H3, I2) noted a lack of clear impact based on patient involvement. “Had we not been in the room, or had we not done the study, would it have made any difference to the appraisal outcome?” (response P7); “Generally, I don’t feel that [patient involvement] has a large impact on decision-making. This may be because of how it is collected” (response I2).

#### Impact on HTA body

This domain covers the impact of patient involvement on the HTA bodies that conduct these assessments, including changes in staff perceptions and adaptations to processes based on patient input.

The most common element under this domain was *HTA staff awareness* about the importance of involving patients in HTA processes (seven mentions) and reminding staff about *HTA’s purpose* (five mentions). “Without patient involvement, we would notably be missing the user’s vision, which is key to the development and implementation of any single treatment” (response H7); patient participation “serves to remind members of the purpose that committees exist to serve – that is, to improve the quality of care” (response H9).

This increased awareness led to a heightened *HTA engagement culture* at the organizational level in four stories, “clear guidance has now been put in place for patient organizations attending meetings” (response H1), and interestingly, to a shift in *HTA perceptions of patients as equal partners* (four mentions). Active patient participation builds trust in the patient community’s ability to contribute meaningfully. One HTA body Patient and Community Advisory Committee representative mentioned how patient participation has “encouraged [HTA body] to move from a focus on ‘extracting perspectives’ to ‘growing relationships’ with patients. Recent projects have involved ‘patient collaborators’ interacting directly with project teams” (response P11).

The *direct contact* through patient participation was also cited as a key element of impact in three stories, “the ability to ask [patient representatives] supplementary questions before the meeting (and after their submission) was really helpful” (response H2).

Lastly, two stories noted that patient representatives suggested *HTA process improvements*; “we fed back that perhaps a video presentation could be considered for future meetings to demonstrate how devices are used” (response H1).

#### Impact on patient participants

This domain covers the impact on the patient representatives participating in HTA.

The most common impact identified under this domain was the feeling of *acknowledgment*, being heard and being valued (three mentions). “We felt our experiences and what mattered had been made somewhat visible and considered as part of the decision-making process… it alleviated a little of the stress and anxiety for me and my community that our voices were not being heard” (response P1); “I had the impression that patient input was valued and that many of the inputs and aspects of the testimonies and arguments put forward were taken up for the final determination” (response P3).

There was also the perception of *co-construction* (three mentions) as patient stakeholders felt as partners in the deliberation process, “patient input is not always about providing information about burden of disease and patient experience. It can often be about being an honest broker and advocating for practical and pragmatic solutions.” (response P4), which led in one case to better *patient acceptance* of the HTA recommendation, “being able to attend a hearing made a difference to how I, and to some extent my community, felt about the decision-making process” (response P1).

Moreover, the ability of patient representatives to participate in HTA processes led to broader *patient awareness about HTA* (one mention) and increased culture of *patient participation in HTA* (two mentions) across the community: “As a patient, I gained a greater understanding of HTA processes, how to have effective input and the need to educate and engage more patients about this process” (response P1).

### Areas for improvement

#### Transparency and expectation-setting

The most significant area for improvement that surfaced was the need to increase *transparency about HTA processes* (nine mentions), including who is invited and how testimonies will be considered: “There was little transparency about the process – which patient groups had been invited and how the information was ‘weighted’ or how information would be valued against the other information that was placed in front of decision makers” (response P1); “The way that patient feedback is reported makes it difficult to know how it impacts decision-making. [HTA bodies] usually provide the names of the organizations submitting feedback, but there is no detail on how it factored into decision-making” (response I2); “the process… needs more clarification on how patient inputs are being considered; it needs criteria” (response P12). The importance of this transparency and its reporting is highlighted by patient organization response P8, “the challenge has always been what is the impact of our activity (we have limited time/resources) and, when cynical, [we] question if the outcome would be any different if we were not involved.”

There was also a call to improve *feedback* as part of the submission process (five mentions), for example, instituting a feedback process or following up post-hearing, “we would appreciate regular follow-ups with updates on actions and progress since the decision; knowing what stage it is currently at or a timeframe for when [procedure] will be introduced to [health system] is important to us and we would appreciate an update” (response P9).

Lastly, patient participants request more *guidance* be shared about the process (two mentions) so they can effectively participate, including type and length of testimony, setting, and audience. “I had little understanding of the hearing itself – the format, length, etc.” (response P1).

#### Systems and processes

*Systematic involvement* of patients across different stages of HTA processes was called for in seven stories, “we operate behind a wall, send a document into a black box, and occasionally get asked to send in a couple of additional documents; never a phone call or meeting to resolve any uncertainties or allow us to address any questions they are wrestling with. The manufacturer has an opportunity to respond to questions during the process, however the patient organization is not afforded any opportunity to give perspective on these uncertainties” (response P5).

There was also a call for a more *systematic review* of patient involvement processes (four mentions), “The analysis of contributions can be more in-depth and consistent from a theoretical and methodological point of view” (response H4); “try and somehow quantify and/or weight the impact on decision-making our participation had beyond just saying that it was really good that you were in the room, and the committee thought you were great” (response P7).

Lastly, the responses surfaced additional opportunities to improve participation, such as *publishing and promoting opportunities* to get involved (three mentions), *increasing the representativeness of patient participants* (three mentions), and *improving submission templates* for patient testimonies (one mention); “questions get repetitive (given frequent submissions) and do not get to the core of our message regarding that submission. We have to work outside the box to comment on what they are NOT asking us… This has made the template more or less useless. We use it as a blank piece of paper” (response P5).

#### Training and data readiness

Seven stories called for *HTA personnel training* to increase the awareness of patient involvement as a core value of HTA, how to incorporate patient insights into their work, and what is appropriate and reasonable to ask patients. “The [HTA] team noted that the most important factors that inhibited the patient involvement process were their lack of experience in previous work with patients and their inexperience using lived knowledge from patients in their prior assessments” (response H7); “more can be done to improve panel member’s confidence in speaking to and asking questions of patients” (response H9). Sensitivity training is especially needed in fostering productive dialogues, “some of the questions asked of me at the hearing were not appropriate and should have been asked of the appropriate subject matter experts rather than myself” (response P1), “sometimes we have to “dumb down” the language [of our submission] because we get feedback that it appears to be professionally written. Some of us are professionals” (response P5).

Four stories suggested *patient training* on the HTA process (what it is, how to contribute) would be necessary to support effective participation, “Patients are likely to have a wealth of experience and feedback that could be valuable, but they just don’t know what information to provide” (response I2). There was also a mention of the usefulness of developing a *buddy system* to support participation, “I was fortunate that the consumer representative was willing to speak with me directly to provide some guidance in how to prepare” (response P1). HTA officials that submitted responses offered specific suggestions about how the patient community could present more *robust data* (four mentions): “more quantitative information on the impact on families outside of QALY gains” (response H2); “more detailed accounts of the effect of [medicine] on patients’ quality of life, more specific patient stories on the transformative effect of the drug, better clarification of wide clinical severity presentation and impact of interpretation of QALY” (response H5).

Lastly, two entries mentioned the importance of decreasing resistance and negative perceptions of patient involvement in HTA amongst *other stakeholders*, for example, the medical community: “We saw some initial resistance from some health care professionals about the value of patient involvement, but most were pleasantly surprised with the experience” (response H7).

## Discussion

Patient involvement in HTA activities contributes to the relevance, fairness, equity, and legitimacy of HTA results and recommendations and, therefore, to the accountability and credibility of the deliberation process (10). As patient involvement takes time and resources from all stakeholders, it must be effective, efficient and minimize the burden to patient communities and HTA bodies. Since 2019, PCIG has collected stakeholder perspectives on the impact of patient involvement in HTA to identify the different facets of impact and encourage reflection on ways to improve patient involvement processes.

This explorative study gathered real-life experiences to assist in characterizing and reporting impact. Responses showed various ways in which patients were involved, from the submission of comments to presentations of perspectives during hearings, to consultations and co-creation. Co-creation included patients participating in workshops, providing input and feedback along the process, and as members of expert advisory committees. The broad range of reported impacts of patient involvement can be categorized under three domains: basis of HTA result or recommendation, HTA body, and patient participants. Examples of impact on the basis of HTA results or recommendations include presenting evidence and perspectives of patient and caregiver needs, cost considerations, preferences, and experiences. Consistent with the literature (11–14), patient stakeholder contributions allowed for contextualized and therefore, better interpretation of the evidence, including validation of existing claims or clarification of patient priorities, which researchers or clinicians may have otherwise interpreted differently. Impact of patient involvement on the HTA body comprised changes in the engagement culture, increased understanding of the value of involving patients, and subsequent process improvements. Impact on the patient stakeholders included higher awareness and understanding of HTA, better decision acceptance, and improved capabilities to identify and express needs in HTA deliberations. All three domains are significant to public health and validate patient involvement as a core competency for HTA bodies (15).

Based on this categorization, the Three-Domain Impact Framework is proposed for identifying, evaluating, and communicating the impact of patient involvement in HTA. Finding appropriate measures for evaluating patient involvement in HTA is not an easy endeavor, and it has been proposed previously that a mix of quantitative and qualitative measures is required (12,16,17,18). The results of this project are particularly beneficial to HTA bodies seeking to improve or develop their evaluation practices. Transparent tracking and reporting within these consistent domains can support ongoing research and stimulate and align awareness of the value of patient involvement among all stakeholders. This can then facilitate a common understanding and general process improvement for more meaningful integration of patient stakeholders into HTA across jurisdictions (3).

More systematic involvement of patients, improving processes, and staff training surfaced as opportunities for improvement by HTA bodies. Patient groups can, in turn, strengthen their participation by clarifying how their involvement can be most impactful, that is, interpretation of data and presentation of patient experience and patient needs – and can also reinforce their submissions following guidance gathered from these stories. The identified areas of improvement are in broad agreement with the literature (19–21). Despite increased attention to patient involvement, little progress has been made in addressing associated barriers. By encouraging the reporting of impact, especially in ways that are relevant to patient organizations, the Framework could facilitate increased transparency and shared understanding of impact.

Some of the limitations of this study will need to be addressed through further research. Additional domains could emerge from stories collected across new geographies and from a broader group of stakeholders, for example, more representation from industry and medical stakeholders involved in assessments could result in additional domains specific to these stakeholders. The research also has limitations in that recall of detail declines over time, and situations may not be comparable. Stories come with the inherent bias that they are self-reported accounts presenting one perspective, with variable levels of details across accounts. Some stories presented detailed accounts with specific examples of impact, whereas others only highlighted areas of impact based on broad examples without concrete detail. Further investigation is needed to validate and expand the codifiers presented in Tables 2 and 3, particularly those that were reported by only a few stories. Nevertheless, the story collection template and the list of codifiers is intended to be a resource for researchers and may serve as a tool for semistructured interviews to help draw out additional, more specific, detail of impact.

## Conclusion

Perceived impact was reported by stakeholders who had experienced patient involvement in HTA using a standard questionnaire with a short set of open-ended questions. Important aspects of impact surfaced across three domains: basis of HTA result and recommendation, HTA body, and patient participants. This Three-Domain Impact Framework adds to the current literature by proposing a simple Framework to consistently identify, evaluate, and report on impact of patient involvement in HTA regardless of the stakeholder perspective.

The questionnaire and code book, validated and applied in this study, are suitable for collecting and analyzing a wide range of perceived impacts from all participating stakeholders. Using these tools as a standard across HTA bodies is recommended to enable the development of more consistent, valued, and valuable processes for evaluating and improving patient involvement in HTA.

## Supporting information

Lopez Gousset et al. supplementary material Lopez Gousset et al. supplementary material

## Supplementary material

The supplementary material for this article can be found at http://doi.org/10.1017/S0266462324000400.

## References

[1] Facey KM, Bedlington N, Berglas S, Bertelsen N, Single ANV, Thomas V. Putting patients at the centre of healthcare: progress and challenges for health technology assessments. Patient. 2018;11:581–9. 30051315 10.1007/s40271-018-0325-5

[2] Gagnon MP, Dipankui MT, DeJean D. Evaluation of patient involvement in HTA. In: Facey KM, Hansen HP, Single ANV, editors. Patient involvement in health technology assessment. Singapore: Springer Nature; 2017. p. 201–213.

[3] Weeks L, Polisena J, Scott AM, Holtorf AP, Staniszewska S, Facey K. Evaluation of patient and public involvement initiatives in health technology assessment: A survey of international agencies. Int J Tech Ass in Health Care. 2017;33:715–723. 10.1017/S0266462317000976 29122048

[4] Kristensen FB, Husereau D, Huić M, Drummond M, Berger ML, Bond K, et al. Identifying the need for good practices in health technology assessment: Summary of the ISPOR HTA Council Working Group Report on Good Practices in HTA. Value in Health. 2019;22(1):13–20. 30661627 10.1016/j.jval.2018.08.010

[5] O’Rourke B, Oortwijn W, Schuller T, The International Joint Task Group. The new definition of health technology assessment: A milestone in international collaboration. Int J Tech Ass in Health Care. 2020;36(3):187–190. 10.1017/S0266462320000215 32398176

[6] Goetghebeur M, Cellier M. Deliberative processes by health technology assessment agencies: A reflection on legitimacy, values and patient and public involvement comment on ‘use of evidence-informed deliberative processes by health technology assessment agencies around the globe.’ Int J Health Policy Manag. 2021;10(4):228–231. 32610794 10.34172/ijhpm.2020.46 PMC8167272

[7] Culyer AJ. Use of evidence-informed deliberative processes – learning by doing; comment on ‘use of evidence-informed deliberative processes by health technology assessment agencies around the globe’. Int J Health Policy Manag. 2020;9(6):263–265. 32613796 10.15171/ijhpm.2019.116 PMC7382906

[8] Single ANV, Facey KM, Livingstone H, Silva A. Stories of patient involvement impact in health technology assessments: A discussion paper. Int J Tech Ass in Health Care. 2019;35(4):266–72. 10.1017/S0266462319000552 31337453

[9] Fereday J, Muir-Cochrane E. Demonstrating rigor using thematic analysis: A hybrid approach of inductive and deductive coding and theme development. Int J Qual Methods. 2006;5(1):80–92.

[10] Values and Quality Standards for Patient Involvement in HT. [Internet]. Health Technology Assessment International: Patient and Citizen Involvement Interest Group: Resources & Materials. 2014 [cited 2024 Feb 3]. Available from: https://past.htai.org/interest-groups/pcig/values-and-standards/

[11] Berglas S, Jutai L, MacKean G, Weeks L. Patients’ perspectives can be integrated in health technology assessments: An exploratory analysis of CADTH Common Drug Review. Res Involv Engagem. 2016;2(1):21. 29062521 10.1186/s40900-016-0036-9 PMC5611639

[12] Livingstone H, Verdiel V, Crosbie H, Upadhyaya S, Harris K, Thomas L. Evaluation of the impact of patient input in health technology assessments at NICE. Int J Tech Ass in Health Care. 2021;37:e33. 10.1017/S0266462320002214 33509314

[13] Menon D, Stafinski T, Dunn A, Short H. Involving patients in reducing decision uncertainties around orphan and ultra-orphan drugs: A rare opportunity? Patient. 2015;8:29–39. 25516506 10.1007/s40271-014-0106-8

[14] Facey K, Boivin A, Gracia J, Hansen HP, Lo Scalzo A, Mossman J, et al. Patients’ perspectives in health technology assessment: A route to robust evidence and fair liberation. Int J Technol Assess Health Care. 2010;70:1518–1526. 10.1017/S0266462310000395 20584364

[15] Bidonde J, Meneses-Echavez JF, Asare B, Chola L, Gad M, Heupink LF, et al. Developing a tool to assess the skills to perform a health technology assessment. BMC Med Res Methodol. 2022;22(1):78. 35313812 10.1186/s12874-022-01562-4 PMC8939100

[16] Gunn CJ, Bertelsen N, Regeer BJ, Schuitmaker-Warnaar TJ. Valuing patient engagement: Reflexive learning in evidence generation practices for health technology assessment. Social Science & Medicine. 2021;280:114048. 34052699 10.1016/j.socscimed.2021.114048

[17] Dukhanin V, Topazian R, DeCamp M. Metrics and evaluation tools for patient engagement in healthcare organization- and system-level decision-making: a systematic review. Int J Health Policy Manag. 2018;7(10):889–903. 30316241 10.15171/ijhpm.2018.43 PMC6186472

[18] Toledo-Chávarri A, Triñanes Pego Y, Rodrigo ER, Roteta NI, Novella-Arribas B, Vicente Edo MJ, et al. Evaluation of patient involvement strategies in health technology assessment in Spain: The viewpoint of HTA researchers. Int J Tech Ass in Health Care. 2020;37(1):e25. 10.1017/S0266462320000586 32914735

[19] Facey KM. Developing the mosaic of patient participation in HTA. In: Facey KM, Hansen HP, Single ANV, editors. Patient involvement in health technology assessment. Singapore: Springer Nature. 2017; 51–66.

[20] Gagnon MP, Tantchou Dipankui M, Poder TG, Payne-Gagnon J, Mbemba G, Beretta V. Patient and public involvement in health technology assessment: Update of a systematic review of international experiences. Int J Technol Assess Health Care. 2021 Feb 5;37:e36. 33541449 10.1017/S0266462321000064

[21] Wortley S, Wale J, Grainger D, Murphy P. Moving beyond the rhetoric of patient input in health technology assessment deliberations. Aust Health Rev. 2017;41(2):170–172. 27224935 10.1071/AH15216

