# Supplementary material for: The three-domain impact framework for characterizing impact of patient involvement in health technology assessment
Source: Int J Technol Assess Health Care. 2024 Nov 11;40(1):e52. doi: 10.1017/S0266462324000400 (PMC11579675; doi:10.1017/S0266462324000400)
Supplement: Lopez Gousset et al. supplementary material [file S0266462324000400sup001.pdf]

HTAi Patient and Citizens Involvement Interest Group

Stakeholder Perspectives of the Impact of Patient Involvement in Health Technology Assessment

PARTICIPANT INFORMATION

Please read through this information and the questionnaire in full before agreeing to take part.

This project aims to better understand the impact of patient involvement in processes used to inform decisions about which medicines and medical services are reimbursed (health technology assessment) for example in <your local HTA body>. This includes scientific advice, assessment, appraisal, and post-HTA data collection. Impact means different things to different people, so we want to understand from your perspective if and what has changed because of patient involvement in these discussions.

Why have I been invited to take part?
You are invited to participate because you experienced patient involvement in the assessment of a medicine, medical service or scientific advice and may have insights about its impact.

How do I take part?
We are interested in your perspective on how patient involvement enriched, supported or impacted the assessment process. We are also interested in learning of instances where patient involvement did not have an impact or where the process for patient involvement could have been improved. As this is your perspective, please describe what you experienced or observed in your own words. We value any details you wish to provide that can help us to better understand your experience, for example you can indicate anything about your condition or your country's processes that might have impacted your experience.

Do I have to take part?
No, this is only an invitation. You are still free to decline participation at any time and without giving a reason, just let us know you do not wish to take part. If you do decide to share your experience, please keep this Participant Information sheet and complete the Participant Consent Form to show that you understand your rights in relation to the project and agree to participate.

Who is doing this study?
An international group of members of the Health Technology Assessment international (HTAi) Patient and Citizen Involvement in HTA Interest Group (PCIG)[1] are leading this project in a voluntary capacity and this work is overseen by the PCIG Steering Group.

What will you do with my completed questionnaire?
These questionnaires will be used to create a series of case studies which can be presented as individual examples or analysed by researchers to better understand the wide range of impact patient involvement has in processes to assess the value of medicines and health services. The results of this project may be summarised in published articles, reports and presentations. Information will be checked with you prior to publication by the HTAi PCIG *(see data protection)*.

What are the possible benefits of taking part?
By sharing your experiences with us, you will be helping HTAi Patient and Citizen Involvement Interest Group understand how patient involvement is impacting the different stakeholders involved in the HTA process and identifying elements that add value and elements that can be improved.

[1] https://htai.org/interest-groups/pcig/

What are the possible risks of taking part?
No significant risks have been identified. Taking part is unlikely to cause distress, but if it does, please advise us. You are free to stop taking part at any time. No reason is required. Please indicate in the survey form if you see any potential for an organisational or occupational risk.

What about data protection?
All completed templates will be kept in a secure password protected system. They will be made available to the Project Team and those undertaking bona fide research approved by the Project Team. We would value including your name when sharing this material so that people can contact you. If you would prefer to remain anonymous, your input will only be shared with the Project Team. However, please note that the details you provide in the template may enable others to identify you once your perspective is shared given information that may be publicly available about the assessment.

How long will it take?
Once you have selected and reflected on the experience you want to share, it should take about 30 minutes to write it up in the questionnaire. If you would prefer to talk, you can also ask to complete the questionnaire verbally and we can arrange a call to discuss your experience.

Who can I contact?
If you have any further questions about the project, please contact:
Anke-Peggy Holtorf, email: HTAi-PCIG@health-os.com

If you wish to make a complaint about the project, please contact:
Ann Single, PCIG Chair, email: singlehaworth@gmail.com

Please sign box
(enter your initials)

	Please sign box *(enter your initials)*
1. I confirm that I have read and understood the Participant Information	
2. I have been given the opportunity to consider the information provided, ask questions and have had these questions answered to my satisfaction.	
3. I understand that my participation is voluntary and that I can ask to withdraw at any time without giving a reason and without penalty.	
4. I understand that my name and organisation (if applicable) will be recorded and the information I provide will be attributed to me unless I mark the box indicating it is to be anonymised.	
5. I understand that the HTAi Patient and Citizen Involvement Interest Group (PCIG) will publish information from my completed template (making it publicly available) and that I will be given the opportunity to check/edit information attributed to me before PCIG publishes it.	
6. I agree to take part in this project.	

Name of person giving consent	Date	Signature
______________________________	_ _/_ _/20_ _	______________________________

Name of person taking consent	Date	Signature
______________________________	_ _/_ _/20_ _	______________________________

STAKEHOLDER PERSPECTIVES OF THE IMPACT OF PATIENT INVOLVEMENT IN HEALTH TECHNOLOGY ASSESSMENT (HTA)

About You

Reported by <your name>: ....

Contact E-Mail:... Phone: ....

Please mark the most suitable box.
These are my:

- ☐ Personal views, or
- ☐ Organisation's views. My organisation is:.........

This information can be published:

- ☐ Attributed to me and checked with me before publication, or
- ☐ Attributed to my organization and checked with me before publication, or
- ☐ Anonymised, so that I am not identified as the source of the story, but I can check the detail before publication.

About the HTA[2] Activity

Country: ....

Date (approx.) when event occurred ....

HTA-or Reimbursement Process / Body: ....

Indication(s): ....

Health Technology[3]: ....

In what capacity are you sharing this information?

- ☐ Patient/health consumer
- ☐ Informal caregiver/carer, e.g. family member
- ☐ HTA staff member
- ☐ Researcher
- ☐ Patient organization representative
- ☐ Industry staff
- ☐ Other *(Please define below)*

How were patient involved in the reimbursement process or health technology assessment (HTA) activity?

For example

- *Patient Group Submissions*
- *Online comments*
- *Meetings (with whom?) or hearings*
- *Focus groups or interviews*
- *Submission of Patient-based evidence / preference data*
- *Presentation to appraisal committee*

If submissions or other materials are publicly available, please provide the links at the end of the questionnaire.

[2] This includes scientific advice, assessment, appraisal, and post-HTA data collection
[3] Medicine, medical device, surgical procedure, vaccine etc; Write n/a if not applicable

Your reflections

Take some time to think about the occasion in which you took part or you observed patients taking part of a reimbursement or health technology assessment process. Did something change as a result? Did patient involvement have an impact on the assessment, your organisation, your community or yourself? We made some suggestions of the types of impact but would appreciate to hear about your experience and perspective in your own words.

What difference did you feel patient involvement made in this specific HTA activity? Can you think of specific ways patient involvement changed the conversation, raised issues which had not been considered or had a material effect on the final opinion? (can be more than one, narrative of experience welcome)

For example

- *Identification of outcomes of importance to patients (including aspects of quality of life)*
- *Identification of groups with high unmet needs*
- *Clarification of patient pathway / journey (including non-therapeutic aspects of disease)*
- *Standard treatment not the same as comparator studied*
- *Challenge / contradict expert held view*
- *Explanation of what the treatment effects (positive and negative) mean in real life*
- *Challenge / contradict expert held view*
- *Explanation of what the treatment effects (positive and negative) mean in real life*
- *Additional evidence that contributed to clinical or cost effectiveness*
- *Cost implications or offsets from patient perspective*
- *Change in HTA recommendation*
- *Learning*
- *Unexpected consequences*

What would have been missed without patient involvement?

What could have been improved in the patient involvement process? Can you think of specific instances where you felt that patient comments were ignored, overruled or set aside? Were there barriers to patient participation?

Additional Remarks

For patients, informal caregivers and patient representatives: How much time was needed to prepare and be involved in this reimbursement or health technology assessment process? (estimate ranges are acceptable)

For HTA bodies or industry: What support did you give to the patients involved?

Have details of the HTA/reimbursement activity been published?
☐ yes ☐ no ☐ I don't know/I haven't been informed
If yes, where: ....
(Please give link to the publication/output related to the HTA activity or suggest someone we may contact who would have this information)

Is there a published report(s) available that documents the patient involvement?

☐ yes ☐ no ☐ I don't know/I haven't been informed

If yes, where: ……………………………………………………………………………….

(Please give the link to where the information has been published or attach the relevant documents or suggest someone we may contact who would have this information)
